# Six Bacterial Vaginosis-Associated Species Can Form an *In Vitro* and *Ex Vivo* Polymicrobial Biofilm That Is Susceptible to *Thymbra capitata* Essential Oil

**DOI:** 10.3389/fcimb.2022.824860

**Published:** 2022-05-04

**Authors:** Aliona S. Rosca, Joana Castro, Lúcia G. V. Sousa, Angela França, Carlos Cavaleiro, Lígia Salgueiro, Nuno Cerca

**Affiliations:** ^1^Laboratory of Research in Biofilms Rosário Oliveira (LIBRO), Centre of Biological Engineering (CEB), University of Minho, Braga, Portugal; ^2^LABBELS –Associate Laboratory , Braga/Guimarães, Portugal; ^3^Faculty of Pharmacy of the University of Coimbra, University of Coimbra, Coimbra, Portugal; ^4^The Chemical Process Engineering and Forest Products Research Centre (CIEPQPF), Department of Chemical Engineering, Faculty of Sciences and Technology, University of Coimbra, Coimbra, Portugal

**Keywords:** bacterial vaginosis (BV), polymicrobial biofilms, alternative therapy, essential oils, microbial interactions

## Abstract

Bacterial vaginosis (BV) is associated with serious gynaecologic and obstetric complications. The hallmark of BV is the presence of a polymicrobial biofilm on the vaginal epithelium, but BV aetiology is still a matter of debate. We have previously developed an *in vitro* biofilm model that included three BV-associated species, but, up to now, no studies are available whereby more bacterial species are grown together to better mimic the *in vivo* situation. Herein, we characterized the first polymicrobial BV biofilm consisting of six cultivable BV-associated species by using both *in vitro* and *ex vivo* vaginal tissue models. Both models revealed that the six species were able to incorporate the polymicrobial biofilm, at different bacterial concentrations. As it has been thought that this polymicrobial biofilm may increase the survival of BV-associated species when exposed to antibiotics, we also assessed if the *Thymbra capitata* essential oil (EO), which has recently been shown to be highly bactericidal against several *Gardnerella* species, could maintain its anti-biofilm activity against this polymicrobial biofilm. Under our experimental conditions, *T. capitata* EO exhibited a high antibacterial effect against polymicrobial biofilms, in both tested models, with a significant reduction in the biofilm biomass and the number of culturable cells. Overall, this study shows that six BV-associated species can grow together and form a biofilm both *in vitro* and when using an *ex vivo* model. Moreover, the data obtained herein should be considered in further applications of *T. capitata* EO as an antimicrobial agent fighting BV.

## Introduction

Bacterial vaginosis (BV) is the worldwide leading vaginal bacterial infection identified in women of childbearing age, with a high prevalence in the general population, ranging from 23% to 29% across regions (Peebles et al., 2019). If left untreated, BV may cause serious obstetric and gynaecologic complications, including preterm delivery (Romero et al., 2004; Shimaoka et al., 2019), spontaneous abortion (Leitich and Kiss, 2007; Işik et al., 2016), low birth weight (Goldenberg and Culhane, 2003; Dingens et al., 2016), pelvic inflammatory disease (Ness et al., 2005; Gondwe et al., 2020), infertility (Salah et al., 2013), and may also lead to an increased risk of acquisition and transmission of several sexually transmitted infectious agents (Gillet et al., 2011; Haddad et al., 2018). Although the understanding of BV etiology is still limited, it is known that BV is characterized by a decrease of beneficial vaginal bacteria and by an increase of strict and facultative anaerobic bacteria (Chen et al., 2021) leading to the development of a polymicrobial biofilm (Swidsinski et al., 2005). This biofilm is thought to protect BV-associated species when exposed to antibiotics, and this leads to recurrent episodes of BV (Bradshaw et al., 2006a; Bradshaw et al., 2012). In addition, the frequent ineffectiveness of standard antibiotics against BV has been also related to high rates of bacterial resistance (Swidsinski et al., 2008; Swidsinski et al., 2011). Consequently, in an attempt to overcome BV treatment failure, natural compounds have been proposed as a promising and effective approach to treat BV (Falconi-McCahill, 2019). Plant-derived products, such as essential oils (EO), represent a therapy on the rise (Stan et al., 2021). EO are complex natural mixtures of volatile compounds produced by aromatic plants, which have been used since ancient times due to their medicinal properties (Bakkali et al., 2008). Furthermore, it has been proposed that the use of EO is associated with a low risk of development of antimicrobial resistance (Becerril et al., 2012; Hammer et al., 2012), which represents a great advantage compared to antibiotics.

*Thymbra capitata* (L.) Cav. [*Coridothymus capitatus* (L.) Rchb. F.] is a circum-Mediterranean plant belonging to the *Lamiaceae* family, widespread in the Mediterranean bay (Verdeguer et al., 2020). This plant is traditionally considered to have strong antiseptic properties, being used for the treatment of cutaneous infections such as acne (Proença-da-Cunha et al., 2014), and in mouthwashes against gum infections (Figueiredo et al., 2008). Several *in vitro* studies have found that *T. capitata* EO exhibited high antimicrobial activity against *Candida* spp. (Palmeira-De-Oliveira et al., 2012), *Listeria monocytogenes* (Faleiro et al., 2005), and *Aspergillus* species (Salgueiro et al., 2004). More important, this EO also showed potent antibacterial activity against *Gardnerella* spp. (Machado et al., 2017). Despite *Gardnerella* spp. prominence, BV is also associated with other bacterial species and, thus, it is of utmost importance to also evaluate their susceptibility to *T. capitata* EO. Therefore, in this study, we characterized, for the first time, a polymicrobial biofilm formed by *G. vaginalis*, *Fannyhessea vaginae* (Swidsinski et al., 2005), *Lactobacillus iners* (Fredricks et al., 2005), *Mobiluncus curtisii* (Fredricks et al., 2007), *Peptostreptococcus anaerobius* (Hillier et al., 1993), and *Prevotella bivia* (Onderdonk et al., 2016), both using an *in vitro* and an *ex vivo* model. We also assessed the susceptibility of these polymicrobial biofilms to *T. capitata* EO, further demonstrating the potential of this natural product as an anti-BV agent.

## Material and Methods

### Plant Material, EO Extraction and Analysis

Aerial parts of *T. capitata* plant were collected at the flowering stage in Carvoeiro, Algarve, Portugal (37°05’33.9”N 8°27’55.5”W). The oil was isolated by hydrodistillation for 3 h from air-dried material, using a *Clevenger*-type apparatus according to the European Pharmacopoeia (Council of Europe, 2010). EO composition was established by the combination of gas chromatography with FID detectors (GC-FID) and gas chromatography-mass spectroscopy (GC/MS) analysis, as previously reported (Sousa et al., 2022). The EO components were identified by considering, concurrently: (i) the acquired retention indices on two columns with different phases, SPB-1 (polydimethylsiloxane) and SupelcoWax-10 (polyethyleneglycol), determined by linear interpolation relative to the retention times of C8–C23 of n-alkanes and compared with reference data from authentic products (available in the laboratory database of the Faculty of Pharmacy, University of Coimbra) and literature data (Wallace, 2021); (ii) the acquired mass spectra compared with reference data from the laboratory database, the Wiley/NIST library (McLafferty, 2009) and literature (Adams, 2007). The relative amount of each component was estimated from GC peaks areas without any correction regarding FID responses.

### Bacterial Species and Growth Conditions

*Gardnerella vaginalis* ATCC 14018 and other five cultivable bacterial species associated with BV were used in the current study, namely *Fannyhessea vaginae* ATCC BAA-55, *Lactobacillus iners* CCUG 28746, *Mobiluncus curtisii* ATCC 35241, *Peptostreptococcus anaerobius* ATCC 27337, and *Prevotella bivia* ATCC 29303. All species were grown as described before (Rosca et al., 2021), on plates containing Columbia Blood Agar Base medium (CBA) (Oxoid, Basingstoke, UK) supplemented with 5% (v/v) defibrinated horse blood (Oxoid) and incubated, for 48 h, at 37°C in the presence of 10% carbon dioxide (*G. vaginalis*) or in anaerobic conditions (the other five BV-associated species) generated by using AnaeroGen sachets (Thermo Fisher Scientific, Gloucester, UK) in sealed jars (Oxoid).

### Minimum Inhibitory Concentration (MIC) and Minimum Lethal Concentration (MLC) Determination

MIC and MLC values of *T. capitata* EO were determined by broth macrodilution method, in glass flasks (McCartney type), as previously performed (Machado et al., 2017), with some minor modifications. Furthermore, MIC values of metronidazole were also determined according to the broth microdilution method, in 96-well tissue culture plates (Orange Scientific, Braine-l’Alleud, Belgium), as done before (Jorgensen and Ferraro, 2009), with a few minor changes. Then, after performing serial dilutions for the EO or metronidazole, using New York City III broth supplemented with 10% (v/v) inactivated horse serum (Biowest, Nuaillé, France) (NYC III), bacterial suspensions of the tested species with an optical density (OD) of ~0.1 (Biochrom EZ Read 800 Plus, Cambridge, UK) at 620 nm were added to the prepared EO or metronidazole dilutions. Subsequently, the glass flasks or the 96-well tissue culture plates were incubated for 48 h at 37°C in anaerobic conditions, as described above. NYC III broth and bacterial suspensions without EO or metronidazole were used as negative and positive controls, respectively. Furthermore, an additional negative control was included for the EO experiment, where dimethyl-sulfoxide (DMSO) (Scharlau, Spain) was added to the bacterial suspension, without EO, and no negative effect was observed on bacterial growth. After 48 h of incubation, the MIC value was determined by reading the OD at 620 nm. MLC was determined by plating 10 µL of each dilution on CBA plates and defined as the lowest concentration of EO that prevented the growth of treated bacteria on agar plates. The MIC and MLC assays were repeated at least three independent times, with technical duplicates for each determination.

### *In Vitro* Biofilm Formation and Quantification

A previously described *in vitro* polymicrobial biofilm formation model (Rosca et al., 2021) was followed in this study, but using six, instead of three BV-associated species. Briefly, the inoculum of each species was grown in NYC III broth and incubated for 24 h at 37°C under anaerobic conditions, as above mentioned. Subsequently, the 24 h cultures were adjusted to a concentration of ~ 1.0 × 10^7^ cells mL^-1^, and the six species were further co-incubated anaerobically in 24-well tissue culture plates (Orange Scientific) for 24 h at 37°C. We used ~1.0 × 10^7^ cells mL^-1^ as starting inoculum for each species, since we have previously demonstrated it to be a good starting concentration, to allow bacterial integration in the polymicrobial biofilms (Castro et al., 2021; Rosca et al., 2021). The final volume in each well was 1 mL. Single-species biofilms were grown as controls. After incubation, the biofilms were washed once with 1 mL 1× phosphate-buffered saline (PBS) and then used for quantification of their biomass and cells culturability. To quantify the biomass of single-species and polymicrobial biofilms, we used the crystal violet (CV) method, as described before (Castro et al., 2021). To further determine the culturability of cells from biofilms, we used the colony-forming units (CFU) method. Therefore, after being washed with 1 mL of 1× PBS, the biofilm in each well of the 24-well plates was scrapped off and resuspended in 1 mL of NYC III broth. Ten-fold serial dilutions were performed in 0.9% (w/v) NaCl (Liofilchem, Roseto degli Abruzzi, Italy) and plated on CBA plates to allow CFU counting. The CBA plates were further incubated anaerobically at 37°C for up to 72 h. At least three independent assays, with technical duplicates, were performed.

### Formation and Characterization of Polymicrobial Biofilms on the Reconstructed Human Vaginal Epithelium

To mimic the vaginal epithelial environment, a reconstructed human vaginal epithelium (SkinEthic™, Episkin, Lyon, France) was used. A 24 h inoculum of each tested species was resuspended in a medium simulating genital tract secretions (mGTS) (Stingley et al., 2014), at a concentration of ~ 1.0 × 10^7^ cells mL^-1^. A mixture containing a 1:1 ratio of each species was dispensed on the SkinEthic™ tissues for colonization. The SkinEthic™ tissues were then incubated anaerobically for 9 h. The SkinEthic™ tissues were then processed as follows. For qPCR quantification, the tissues were washed once with 0.9% (w/v) NaCl and then processed as described below. To perform the culturability assays, the SkinEthic™ tissues were washed once with 0.9% (w/v) NaCl and 500 µL of mGTS was added before performing serial dilutions and plating them on CBA plates. A cycle of sonication for 10 sec with an amplitude of 33% was performed to displace the cells from the SkinEthic™ tissues. For microscopic analysis, the SkinEthic™ tissues were placed in 4% paraformaldehyde (Thermo Fisher Scientific) and then embedded in paraffin (Leica TP1020, Nussloch, Germany). Paraffin tissue blocks were prepared (Leica EG 1140 H, Nussloch, Germany) and 3-μm-thick sections were obtained using a microtome (Microm HM 325, Walldorf, Germany). For the deparaffinization step, sections were placed in xylene (Thermo Fisher Scientific) twice, followed by a hydration step with 100% and 50% of ethanol (Thermo Fisher Scientific) and a final step in distilled water. All steps were performed for 5 min. Samples were then stained with Periodic Acid-Schiff (PAS), as described before (Chawla et al., 2017). The samples were analysed using an Olympus BX51 microscope (Olympus, Lisbon, Portugal). The experiment was performed in duplicate and repeated three independent times.

### Characterization of *In Vitro* and *Ex Vivo* Polymicrobial Biofilms by Quantitative PCR (qPCR) Quantification

Genomic DNA (gDNA) from the *in vitro* and *ex vivo* polymicrobial biofilms as well as from pure cultures of the six species (for the qPCR calibration curves), were performed as described before (Cerca et al., 2022). To assess the efficiency and variability of gDNA extraction between samples, 10 µL of luciferase complementary DNA (cDNA), obtained as described before (Magalhães et al., 2019), was added to each sample before proceeding with gDNA isolation. Specific primers were designed with CLC genomics workbench version 21 (QIAGEN), and primer specificity was first confirmed using Primer-BLAST and then experimentally determined by qPCR. Primers are described in **Table 1**. The qPCR runs were performed in a CFX96™ thermal cycler (Bio-Rad, CA, USA) with the following cycle parameters: 95°C for 3 min, followed by 40 cycles of 95°C for 5 s, and 60°C for 20 s. Non-template controls were used to define the negative threshold to be considered for each primer set. Melt analysis was performed to ensure the absence of unspecific products and primer-dimers. PCR amplification efficiency was determined from the slope of a standard curve. Bacterial load in each sample was interpolated from the averaged standard curves. All assays were repeated at least three independent times with three technical replicates.

**Table 1:** Specific primers for the quantification of the six bacterial species present in the polymicrobial biofilm.

Target genome	Genomic amplification region	Forward primer	Reverse primer	Amplicon	Amplification efficiency
*Gardnerella vaginalis* ATCC 14018	locus_tag=GAVG_1017 (product=glucose-6-phosphate isomerase)	CAACGGTATCCTGACCGTCT	CCTTGCAAAGGCAGTTAAGC	155 bp	82%
*Fannyhessea vaginae* FDAARGOS934	locus_tag=I6G91_00565 (product=PTS sugar transporter subunit IIB)	CCTCATGCAAAATGTGATGC	CCAAAACAGAAGCACGGAAT	211 bp	80%
*Peptostreptococcus anaerobius* NCTC11460	locus_tag=NCTC11460_00716 (product=Phosphoenolpyruvate-protein phosphotransferase)	TGCAAAGCACGTTGATTTCT	CCGCACATACCTACCCACTT	183 bp	85%
*Mobiluncus curtisii ATCC 43063*	locus_tag= HMPREF0573_10085 (product= putative hydrolase)	AGCACTATTGCCFCTTGATT	TGAGATTTCTTCCGGACCAC	222 bp	84%
*Prevotella fusca JCM 17724^#^*	locus_tag= ADJ77_02570 (product=endolytic transglycosylase MltG)	GGCTGATGAATATGCCTACGA	AAACACCATGTTGCGAATAGC	236 bp	85%
*Lactobacillus iners* KY	locus_tag=GYK47_01550 (product= peptidase M64)	AAAGATCGGCGTATGATTGC	GATTCAACACAGCACTAATT	243 bp	93%
*Exogenous control (Luciferase)*	N/A	TACAACACCCCAACATCTTCGA	GGAAGTTCACCGGCGTCAT	67 bp	100%

^#^Due to the lack of annotated P. bivia genomes at NCBI, we used a conservative region of a similar Prevotella species for primer design, that was later confirmed in vitro.

### The Effect of EO on *In Vitro* and *Ex Vivo* Biofilms Biomass and Biofilm Cells Culturability

For the *in vitro* model, the 24 h biofilms were exposed to 1 mL of *T. capitata* EO at a concentration of 0.63 µL mL^-1^, diluted in NYC III broth containing DMSO, and further incubated anaerobically at 37°C for an additional 24 h. DMSO had the role to improve the solubility of EO in the culture medium. The biomass and cell culturability of the *in vitro* biofilms treated with EO were quantified as described above. For each experiment, initial and final controls, respectively, 24 h and 48 h biofilms were included. The 48 h controls consisted of biofilms to which 1 mL of fresh culture medium without EO was added. For the *ex vivo* SkinEthic™ model, EO challenge at a concentration of 0.32 µL mL^-1^ in mGTS was performed after 9 h of biofilm incubation and allowed to act for 14 h. Total incubation and EO exposure time was limited to a maximum of 24 h in order to follow the manufacturer’s instructions for guaranteeing SkinEthic™ tissue viability. Controls included SkinEthic™ tissues not exposed to the EO. Biofilms biomass was qualitatively determined by optical microscopy, and cell culturability was determined by CFU plating, as mentioned above. The CFU detection limit was log 3 CFU mL^-1^.

### Statistical Analysis

All data were analysed with GraphPad Prism version 7 (La Jolla, CA) using two-way ANOVA with Dunnett’s or Tukey’s multiple comparisons tests. Values with a *p* < 0.05 were considered statistically significant.

## Results

### Ability of the Six BV-Associated Species to Form an *In Vitro* and *Ex Vivo* Polymicrobial Biofilm

**Figure 1:**
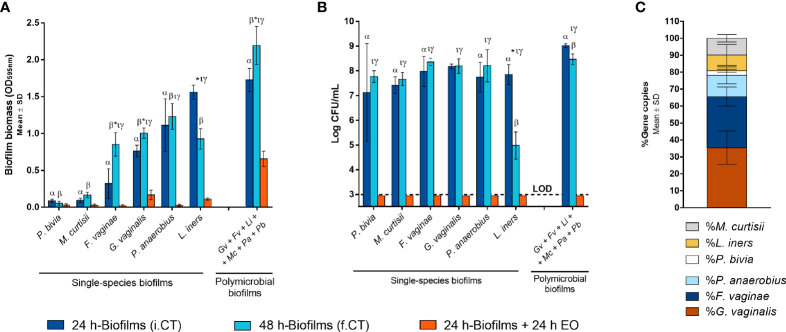
Single-species and polymicrobial BV biofilms. **(A)** Biomass quantification of the single-species and polymicrobial biofilms prior to and after EO exposure using crystal violet method. **(B)** Culturable cells quantification from the single-species and polymicrobial biofilms prior to and after EO treatment, by CFU method. **(C)** Determination of the percentage distribution of each bacterial species in 24 h polymicrobial BV biofilm, by qPCR. i.CT stands for initial control, before the medium replacement; f.CT stands for final control, after incubation with fresh medium. Statistically significant differences between polymicrobial *vs* single-species biofilms are represented with ^α^ for 24 h incubation time and with ^β^ for 48 h incubation time (two-way ANOVA and Dunnett’s multiple comparisons test, *p* < 0.05). Values are significantly different for ^*^ i.CT *vs* f.CT, ^τ^ i.CT *vs* EO, and ^γ^ f.CT *vs* EO (two-way ANOVA and Tukey’s multiple comparisons test, *p* < 0.05). *Gardnerella vaginalis* (*Gv*); *Fannyhessea vaginae* (*Fv*); *Lactobacillus iners* (*Li*); *Mobiluncus curtisii* (*Mc*); *Peptostreptococcus anaerobius* (*Pa*); *Prevotella bivia* (*Pb*); Essential oil (EO); Limit of detection (LOD).

**Figure 2:**
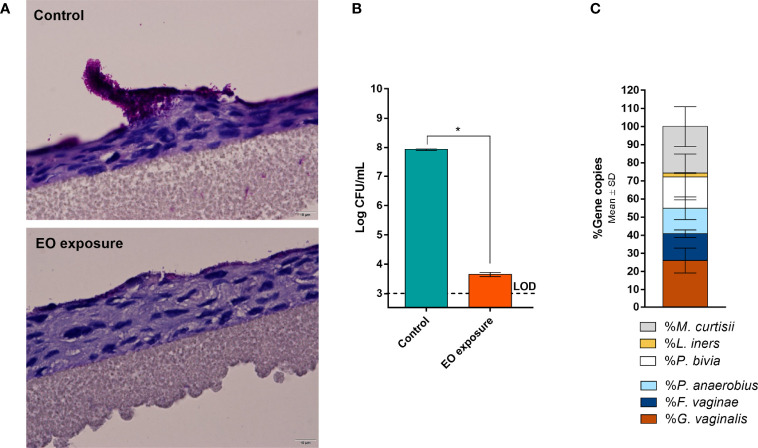
Polymicrobial BV biofilm developed on a reconstructed human vaginal epithelium. **(A)** Periodic Acid-Schiff staining images with the BV biofilm before and after EO treatment at 600× magnification. **(B)** Cells culturability from the control and EO treated biofilms as determined by CFU method. **(C)** Bacterial distribution in the polymicrobial BV biofilms as determined by qPCR after extraction of genomic DNA of each bacterial species. ^*^Values are significantly different for control vs EO-exposed biofilms (paired *t*-test, *p* < 0.05). Essential oil (EO); Limit of detection (LOD). More examples are shown in **Supplementary Material**.

Although BV is associated with the presence of a polymicrobial biofilm on the vaginal epithelium (Swidsinski et al., 2005), up to date, no functional *in vitro* studies have assessed BV-associated biofilms composed of more than three different species (Rosca et al., 2021). Since *in vivo* evidence supports the role of more species to be present during BV, in this study, we aimed to assess if six relevant BV-associated species could, in fact, establish a polymicrobial biofilm. As shown in **Figure 1A**, total biofilm biomass of the polymicrobial biofilms was significantly higher than the biomass of any tested single-species biofilms (*p* < 0.05). Taking into consideration that each species can contribute differently to biomass accumulation, we also determined the total number of culturable cells, which was also higher in the polymicrobial biofilms, as shown in **Figure 1B**. This suggests that synergistic interactions were present during the formation of this biofilm. In order to see if this phenomenon also occurs in conditions more similar to the *in vivo* situation, we further used an *ex vivo* model. As can be observed in **Figure 2A**, the BV-associated species used in this study were also able to form a prominent biofilm structure on the SkinEthic™ tissues.

Importantly, to support our claims that all six species were present after the biofilm incubation period, we determined the biofilm composition after removal of the bulk liquid, by performing gDNA extraction followed by qPCR quantification. In both models, all six species were found inside the biofilm structure, although with slightly different cell percentages between the models (**Figures 1C** and **2C**). It is worth mentioning that the *ex vivo* model experimental conditions were distinct from the *in vitro*, and these two models were used with the goal to further support our conclusions. Due to the different experimental conditions, these two models are not meant to be directly compared.

### Susceptibility of BV-Associated Species Planktonic Cells to EO and Metronidazole

The EO used herein was obtained with a yield of 2.7% (v/w). It is characterized by a high percentage of oxygen-containing monoterpenes, being mainly composed of carvacrol (73.9%) followed by its biogenetic precursors, γ-terpinene (7.4%), and p-cymene (4.9%). The full composition of this EO has been described before (Sousa et al., 2022). *In vitro* antibacterial activity of this EO against each species tested in the current study was evaluated by determining both MIC and MLC values. For comparative purposes, the MIC of metronidazole, the first-line treatment for BV (Workowski and Bolan, 2015), was also determined. As shown in **Table 2**, four out of six species were not susceptible to metronidazole, including *G. vaginalis* and *F. vaginae*, that have been shown to constitute the majority of the biofilm biomass (**Figure 1C**). Furthermore, the EO exhibited a variable antimicrobial effect against BV-associated bacteria, with a concentration of 0.63 µL mL^-1^ inhibiting the growth of *P. anaerobius*, while a concentration of 0.16 – 0.31 µL mL^-1^ being already sufficient to inhibit the growth of *F. vaginae* and *M. curtisii*.

**Table 2:** Determination of the minimum inhibitory (MIC) and minimum lethal (MLC) concentrations of *Thymbra capitata* essential oil against BV-associated species and comparison with standard antibiotics.

Bacteria	EO MIC (µL mL^-1^)	EO MLC (µL mL^-1^)	Metrodinazole MIC^#^ (µg mL^-1^)
*Gardnerella vaginalis* ATCC 14018	0.31	0.63	[8 - 32]
*Fannyhessea vaginae* ATCC BAA-55	[0.16 - 0.31]	[0.31 - 0.63]	>128
*Lactobacillus iners* CCUG 28746	0.31	[0.31 - 0.63]	>128
*Mobiluncus curtisii* ATCC 35241	[0.16 - 0.31]	[0.31 - 0.63]	>128
*Peptostreptococcus anaerobius* ATCC 27337	0.63	0.63	[2 - 8]
*Prevotella bivia* ATCC 29303	0.31	[0.31 - 0.63]	[4 - 8]

^#^The microbiological susceptibility and resistance breakpoints for metronidazole (≤ 8 μg mL^-1^ and ≥ 32 μg mL^-1^) were used as defined by CLSI.

### Impact of EO on Biofilms Biomass and Biofilm Cells Culturability

Despite BV has been associated with a polymicrobial biofilm, we first tested the EO antimicrobial activity against single-species biofilms, for comparative purposes. As shown before (Rosca et al., 2021), the ability to form single-species biofilms varied significantly between species, with *P. bivia* and *M. curtisii* forming negligible biofilms after 24 h of incubation. Interestingly, when using the highest MLC concentration determined for the selected species (0.63 µL mL^-1^), most biofilms biomass was eradicated, except for *G. vaginalis* and *L. iners* (**Figure 1A**). On the polymicrobial biofilm, higher biomass remained after the EO challenge, but when analysing the total culturable cells of the remaining biofilms, no bacteria were quantified in any of the single-species or polymicrobial biofilms (**Figure 1B**). To better assess the EO antimicrobial potential against a BV polymicrobial biofilm, we also used an *ex vivo* model, wherein a polymicrobial biofilm was established on a reconstructed human vaginal epithelium. When applying a lower (0.32 µL mL^-1^), but non-cytotoxic concentration of the EO (Sousa et al., 2022), a significant reduction of the biofilm biomass was also observed (**Figure 2A**), similar to what occurred in the *in vitro* experiment (**Figure 1A**). However, contrary to the *in vitro* experiment, EO exposure did not completely eradicate culturable cells, with only a 4-log reduction observed (**Figure 2B**). However, both the EO exposure periods (24 vs 14 h) and the EO concentrations (0.63 vs 0.32 µL mL^-1^) were different, a consequence of the limitation of the *ex vivo* model, in order to maintain the reconstructed human vaginal epithelium viable.

## Discussion

It is already known that BV is associated with the presence of a polymicrobial biofilm on the vaginal epithelium (Swidsinski et al., 2005). Despite this knowledge, little is known about how this biofilm is formed and what kind of interactions might occur between bacterial species forming it. While direct evidence of *Gardnerella* spp. and *F. vaginae* co-existing in the same biofilm have already been described *in vivo* (Swidsinski et al., 2005), the presence of the other species has mostly been derived from microbiome studies (Fredricks et al., 2005; Fredricks et al., 2007). As such, there is interest in trying to determine if these species can, in fact, interact and grow together in a biofilm. Aiming to better determine the role/impact of bacterial biofilms in BV, biofilm studies have been reported, wherein single- (Janulaitiene et al., 2018; Khan et al., 2019; Li et al., 2020), dual- (Castro et al., 2019; Castro et al., 2020) or triple-species (Rosca et al., 2021) *in vitro* BV-associated biofilms have been characterized. By providing direct evidence that the same biofilm contains the six tested BV-associated species, we are the first to show that close cooperation between these species can occur, at least *in vitro* and in an *ex vivo* vaginal model, as discussed next.

The six species in this study were not randomly selected. They have been commonly used in BV-related microbiome studies (Rosca et al., 2020) and have been previously reported as potential pathogens involved in BV development (Ravel et al., 2011; Onderdonk et al., 2016; Diop et al., 2019). Of note, while all six species were inoculated at a 1:1 ratio, we could not claim that after the incubation period, all six species incorporated the biofilm. Indeed, cumulating evidence from experimental and metabolic model-based studies demonstrated that often microorganisms compete for limited resources, such as space and nutrients (Freilich et al., 2011; Foster and Bell, 2012), at times leading to exclusion. As no specific fluorescence *in situ* hybridization (FISH) probes exist for all six species, we have characterized the ecological niche of this polymicrobial biofilm using qPCR (Bordagaray et al., 2021). As demonstrated by our results, all six species were able to incorporate the biofilm, either using the *in vitro* or *ex vivo* models. Still, some interesting differences were observed between these models, with *P. bivia* and *M. curtisii* having, respectively, a 6.4- and 2.6-fold higher prevalence in the *ex vivo* model, while *L. iners* and *F. vaginae* had a 4.3- and 2.0-fold higher prevalence in the *in vitro* model, respectively. *G. vaginalis* and *P. anaerobius* were the two less affected species. These differences are not surprising, since both the adhesion surface, the growth media, and the incubation time (24 h vs 9 h) used were very different, and these are known to affect biofilm formation ability (Tolker-Nielsen, 2015). The choice of mGTS for the *ex vivo* model aimed to better mimic *in vivo* conditions. While the *ex vivo* model still lacks some relevant components, such as the presence of host immune components (Castro et al., 2018; Delgado-Diaz et al., 2020) and human hormones (Moncla et al., 2016), the observed biofilm biomass was similar to what has been described *in vivo* (Hardy et al., 2016). As functional studies are not possible to perform *in vivo*, these *in vitro* and *ex vivo* models are fundamental to better understand the aetiology of BV, despite the limitations.

Once formed, the presence of this polymicrobial BV biofilm leads to negative consequences for women's health as it is considered one of the major factors responsible for the treatment failure. This may be due to the fact that this biofilm could provide protection and allow BV-associated bacteria to display increased tolerance to antibiotics, and this may be further contributing to high rates of BV recurrence (Vodstrcil et al., 2021). To overcome this problem, alternative approaches to existing antibiotics are being currently pursued (Sherrard et al., 2018; Marrazzo et al., 2019). Herein, we further explore the potential of *T. capitata* EO against BV. We have previously shown *T. capitata* EO to be highly effective against *Gardnerella* spp. single-species biofilms and non-toxic for several lactobacilli (Machado et al., 2017) while also not being toxic for the reconstructed human vaginal epithelium (at the used concentrations) (Sousa et al., 2022). In the present study, the EO showed high antimicrobial activity at MLC concentration against the single-species biofilms. This was specially relevant because most of the tested species were resistant to metronidazole. These results are very encouraging as several previous studies have also shown that BV-associated species are often resistant to antibiotics (Ferris et al., 2004; Bahar et al., 2005; Bradshaw et al., 2006b; Alves et al., 2014; Castro et al., 2015). Furthermore, our data also showed that EO at MLC concentration had a significant reduction effect on the total biomass of polymicrobial biofilms and this should be considered in further applications of this EO as an antimicrobial agent.

Taken together, this brief report brings novelty to the BV research field as (i) this is the first time six BV-associated species have been reported to establish an *in vitro* biofilm; (ii) this is the first time such biofilm is shown to be formed on a reconstructed human vaginal epithelium using an *ex vivo* model; and (iii) this is the first study to demonstrate the antimicrobial activity of *T. capitata* EO against some prominent bacterial species associated with BV, in either pure cultures or in a six-species biofilm consortium. Nonetheless, this study has some limitations and further work should be performed in order to address (i) the possible presence of viable but non-culturable cells and (ii) the composition of the biofilm after EO exposure, by developing a method that allows discrimination between live and dead cells, such as viability PCR (Latka et al., 2022) or FISH (Castro et al., 2022). As we are already heading toward a post-antibiotic era in which many bacterial infections will be impossible to treat (Hauser et al., 2016), it is of utmost importance to focus attention on new antimicrobial treatment options, and *T. capitata* EO could be an ideal candidate in this regard.

## Data Availability Statement

The original contributions presented in the study are included in the article/**Supplementary Material**. Further inquiries can be directed to the corresponding author.

## Author Contributions

LS collected the plants; LS and CC extracted and characterized the EO; ASR performed the planktonic and biofilm experiments. JC and LGVS performed the experiments with the reconstructed human vaginal epithelium. AF and NC performed the qPCR experiments. NC designed the study. ASR and NC drafted the manuscript. All authors critically reviewed and approved the final manuscript.

## Funding

This work was supported by the Portuguese Foundation for Science and Technology (FCT) by the research project [PTDC/BIA-MIC/28271/2017] under the scope of COMPETE 2020 [POCI-01-0145-FEDER-028271] and by the strategic funding of unit [UID/BIO/04469/2020]. ASR acknowledges the support of FCT individual fellowship [PD/BD/128037/2016]. The founder had no role in the study design and collection, analysis, and interpretation of the results.

## Conflict of Interest

This work has been submitted as part of a patent request.

## Publisher’s Note

All claims expressed in this article are solely those of the authors and do not necessarily represent those of their affiliated organizations, or those of the publisher, the editors and the reviewers. Any product that may be evaluated in this article, or claim that may be made by its manufacturer, is not guaranteed or endorsed by the publisher.
