# Supplementary material for: Six Bacterial Vaginosis-Associated Species Can Form an In Vitro and Ex Vivo Polymicrobial Biofilm That Is Susceptible to Thymbra capitata Essential Oil
Source: Front Cell Infect Microbiol. 2022 May 4;12:824860. doi: 10.3389/fcimb.2022.824860 (PMC9114774; doi:10.3389/fcimb.2022.824860)
Supplement: Supplementary file 1 [file DataSheet_1.pdf]

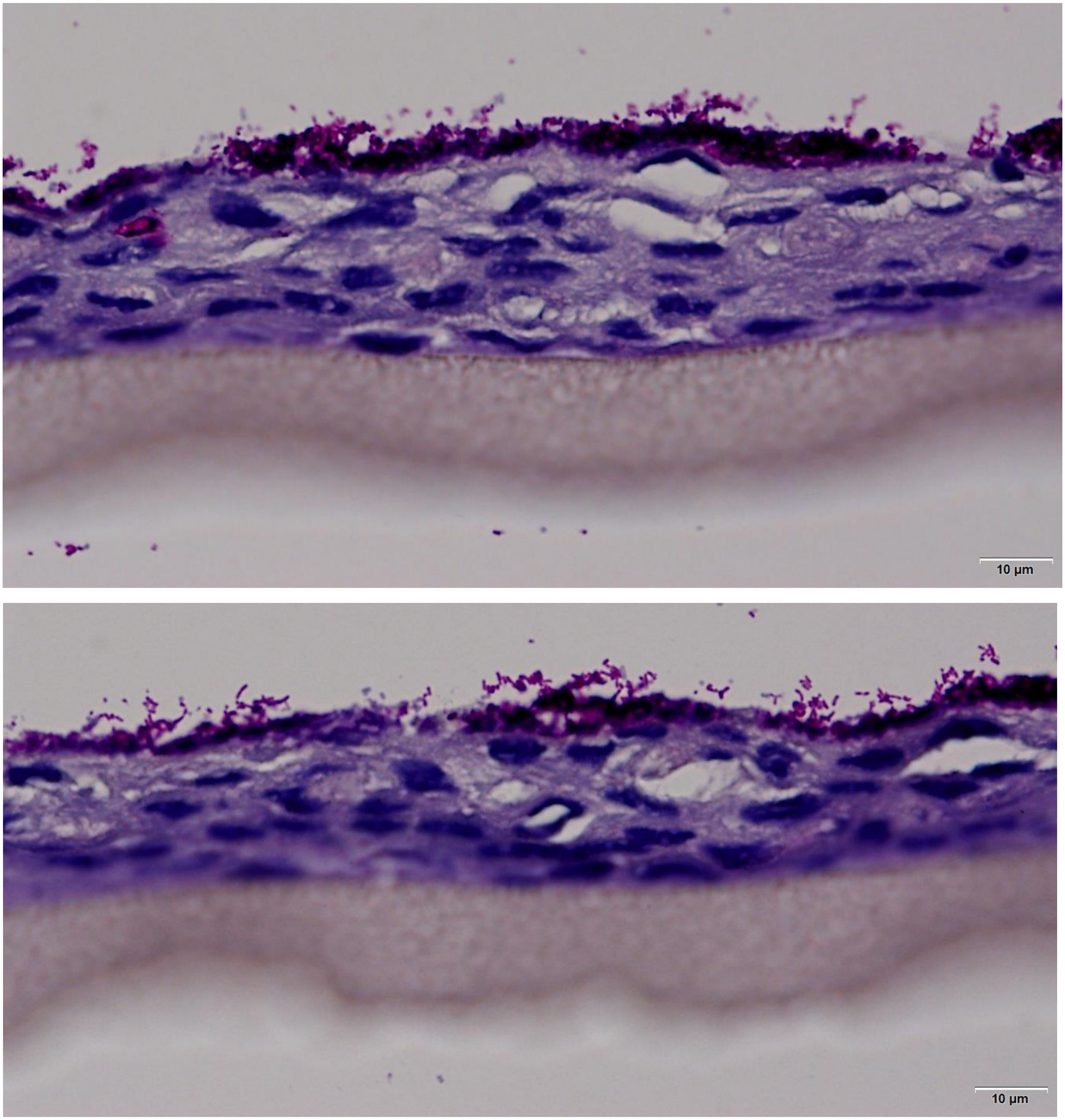

Supplementary Figure 1. An example data set on polymicrobial BV biofilm developed on reconstructed human vaginal epithelium. Periodic Acid-Schiff staining images with BV biofilm before EO treatment at 600× magnification.

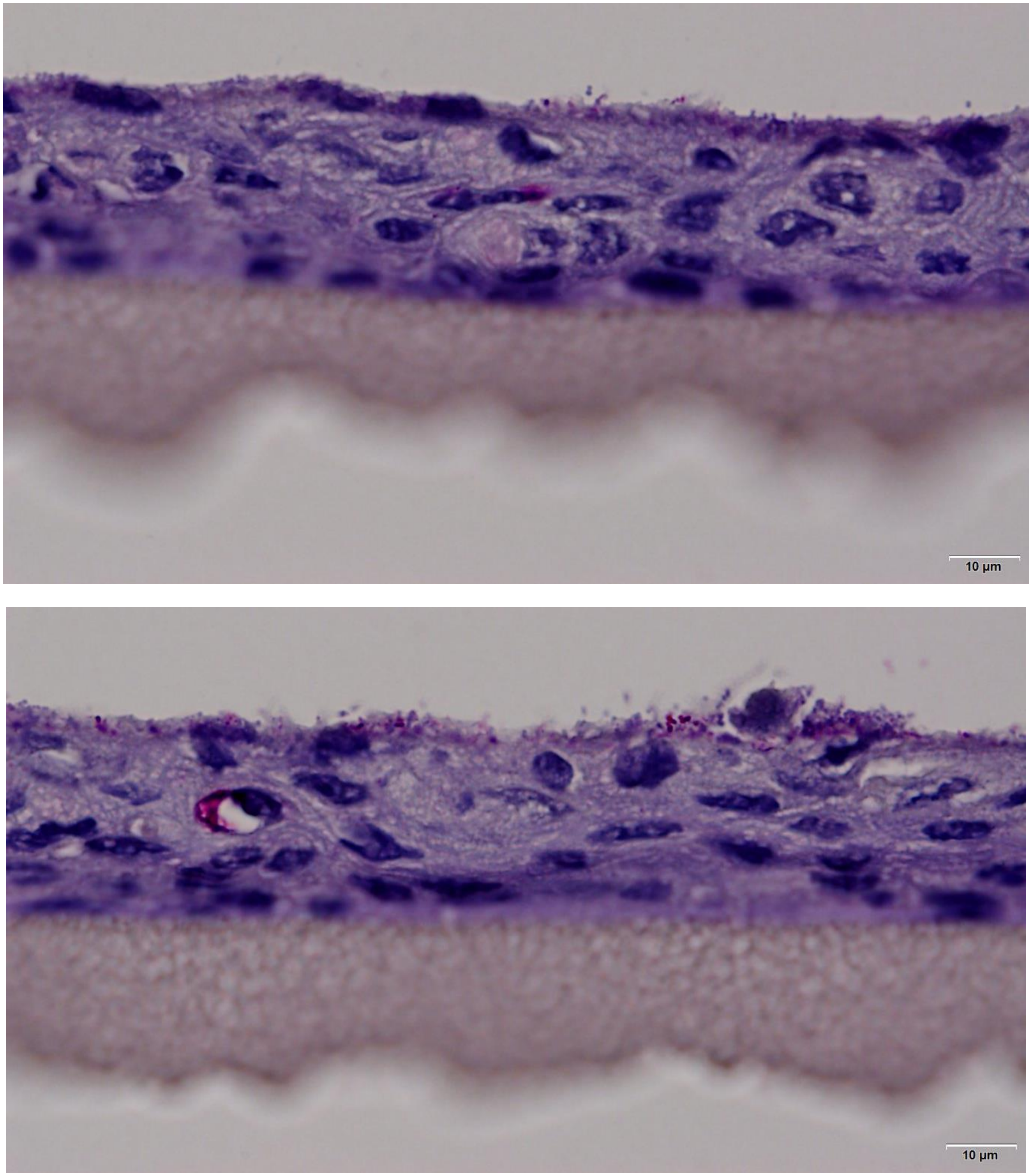

Supplementary Figure 2. An example data set on polymicrobial BV biofilm developed on reconstructed human vaginal epithelium after EO treatment. Periodic Acid-Schiff staining images at 600× magnification.
